#  Fatal Case of Splash Pad–Associated *Naegleria fowleri* Meningoencephalitis — Pulaski County, Arkansas, September 2023

**DOI:** 10.15585/mmwr.mm7410a2

**Published:** 2025-03-27

**Authors:** Theresa M. Dulski, Forrest Montgomery, Jeanette M. Ramos, Eric R. Rosenbaum, Bobby L. Boyanton, Courtney M. Cox, Steven Dahl, Cole Kitchens, Terry Paul, Amy Kahler, Alexis Roundtree, Mia Mattioli, Michele C. Hlavsa, Ibne K. Ali, Shantanu Roy, Julia C. Haston, Naveen Patil

**Affiliations:** ^1^Career Epidemiology Field Officer Program, CDC; ^2^Arkansas Department of Health; ^3^University of Arkansas for Medical Sciences, Little Rock, Arkansas; ^4^Arkansas Children’s Hospital, Little Rock, Arkansas; ^5^Division of Foodborne, Waterborne, and Environmental Diseases, National Center for Emerging and Zoonotic Infectious Diseases, CDC.

**Summary**

**What is already known about this topic?**

Most *Naegleria fowleri* infections are life-threatening and associated with swimming or diving in fresh water, such as a lake. During 2020–2021, two fatal infections associated with splash pads (interactive water play venues that spray or jet water on users) were reported to CDC.

**What is added by this report?**

In September 2023, a fatal splash pad–associated *N. fowleri* infection in a young child occurred in Arkansas. An investigation identified inadequate disinfection of splash pad water.

**What are the implications for public health practice?**

Splash pads with inadequately disinfected water are an emerging exposure of concern for *N. fowleri* transmission. Infection should be considered in patients with acute meningoencephalitis and history of recent exposure to fresh water, including treated recreational water (e.g., in splash pads or pools). Proper design, construction, operation, and management of splash pads can help prevent transmission of pathogens, including *N. fowleri*.

## Abstract

A fatal case of primary amebic meningoencephalitis (PAM), an infection caused by *Naegleria fowleri*, was diagnosed in Arkansas in a young child in September 2023. A public health investigation was completed, with epidemiologic, laboratory, and environmental data suggesting that a splash pad (an interactive water play venue that sprays or jets water on users and has little or no standing water) with inadequately disinfected water was the most likely site of the patient’s *N. fowleri* exposure. This case is the third occurrence of splash pad–associated PAM reported in the United States; all three cases involved inadequately disinfected water. PAM should be considered in patients with acute meningoencephalitis and a history of recent possible exposure to fresh water, including treated recreational water (e.g., in splash pads or pools), via the nasal passages. Proper design, construction, operation, and management of splash pads can help prevent illnesses, including *N. fowleri* infections. Increased awareness, collaboration, and communication among clinicians, hospitals, laboratories, CDC, health departments, the aquatics sector, and the public can help support *N. fowleri* infection identification, treatment, prevention, and control efforts.

## Case Identification and Clinical Course

On September 1, 2023, a previously healthy child aged 16 months was seen at a local Arkansas hospital with a 3-day history of fever, vomiting, decreased oral intake, decreased activity, and new onset of altered mental status. A noncontrast head computed tomography scan demonstrated ventriculomegaly, a sign concerning for increased intracranial pressure, a possible sequela of meningitis. Empiric treatment for bacterial and viral meningitis was initiated. The patient was admitted to the pediatric intensive care unit for further evaluation and treatment. A lumbar puncture was performed, and findings on laboratory examination of cerebrospinal fluid (CSF) were consistent with possible bacterial meningitis.^§^ Qualitative multipathogen nucleic acid–based CSF test results (bioMérieux BioFire FilmArray Meningitis/Encephalitis Panel) were negative; blood and CSF cultures demonstrated no growth after 24 hours. The patient’s clinical condition worsened.

**FIGURE 1:**
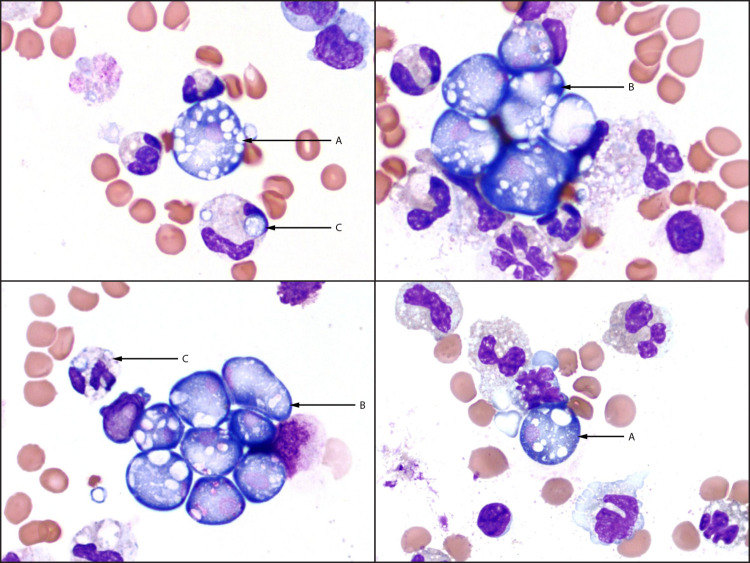
Wright-Giemsa stained (x1,000 magnification) cerebrospinal fluid cytospin slide images from a patient with fatal primary amebic meningoencephalitis, demonstrating numerous *Naegleria fowleri* trophozoites seen as extracellular single forms (A) or clusters (B), with a predominantly neutrophilic background inflammatory response including neutrophils phagocytizing *N. fowleri* microorganisms (C) — Pulaski County, Arkansas, September 2023 Photos/Jeanette M. Ramos, Arkansas Children’s Hospital Department of Pathology

On September 3, pathology review of Wright-Giemsa stained cytospin slides of CSF revealed numerous amebic microorganisms morphologically consistent with *Naegleria* spp*.,* a free-living ameba that is found in warm fresh water and soil and causes primary amebic meningoencephalitis (PAM) (Figure 1) (*1*). Family members reported that the patient had played at a splash pad and pool in Pulaski County, Arkansas, on August 26 and 27 (2–3 days before symptom onset). Treatment for *Naegleria fowleri* infection (amphotericin B, azithromycin, dexamethasone, fluconazole, miltefosine, and rifampin) was started. CDC was consulted, and the case was reported to the Arkansas Department of Health (ADH). CSF specimens were sent to CDC’s Free-Living and Intestinal Amebas (FLIA) Laboratory for further testing. The patient’s condition did not improve, and the patient died on September 4. On September 6, CDC confirmed *N. fowleri* in the child’s CSF via real-time polymerase chain reaction (PCR) testing.

## Public Health Investigation and Response

CDC supported ADH in the case investigation and response. This activity was reviewed by CDC, deemed not research, and was conducted consistent with applicable federal law and CDC policy.^¶^

### Splash Pad and Pool Site Investigation

**FIGURE 2:**
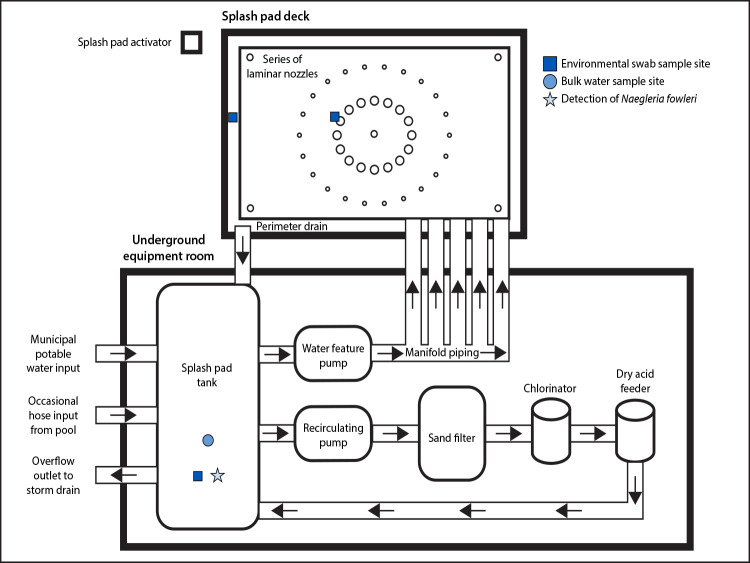
Schematic representation of splash pad water system — Pulaski County, Arkansas, September 2023* * Includes a recirculating system and a separate water feature pump, which pulled water from an underground tank when users activated the splash pad and directed it through a manifold piping system to a series of laminar nozzles that sprayed water up from the deck. After the water fell onto the splash pad, it flowed down a sloped surface into a return drain lining the perimeter and was plumbed back into the tank. A recirculating pump then pulled water from this tank and sent it through a sand filter and, subsequently, calcium hypochlorite tablets and sodium bisulfate tablets were added by a chlorinator and dry acid feeder, respectively. The water was then returned to the tank until being recirculated again, or until a user activated the splash pad again. The splash pad water source was municipal potable water; in addition, water was also occasionally pumped via hose directly from the pool into the splash pad tank. Overflow outlet piping drained from the splash pad tank to a storm drain and helped prevent the tank from flooding.

On September 3, 2023, an ADH environmental health specialist conducted a site investigation of the splash pad and adjacent pool where the patient had played 2–3 days before symptom onset. The splash pad (Figure 2) used a recirculating system and a separate water feature pump, which pulled water from an underground tank when users activated the splash pad and directed it through a manifold piping system to a series of laminar nozzles that sprayed water up from the deck. After the water fell onto the splash pad, it flowed down a sloped surface into a return drain lining the perimeter and was plumbed back into the tank. A recirculating pump then pulled water from this tank and sent it through a sand filter and, subsequently, calcium hypochlorite tablets and sodium bisulfate tablets were added by a chlorinator and dry acid feeder,** respectively. The water was then returned to the tank until being recirculated again, or until a user activated the splash pad again. The splash pad water source was municipal potable water; in addition, water was also occasionally pumped via hose directly from the pool into the splash pad tank. Overflow outlet piping drained from the splash pad tank to a storm drain and helped prevent the tank from flooding.

Multiple code violations related to the splash pad were noted. The pH exceeded the test kit measurement limit of 8.2^††^; the residual chlorine concentration exceeded the test kit limit of 5 ppm^§§^; the chlorinator was reported as nonfunctional for approximately 1 month, and chlorine was being hand-fed daily^¶¶^; no daily operational records were maintained or available for review.***

The pool used a separate, similarly laid out recirculating system that also included a surge tank.^†††^ Multiple code violations related to the pool were also noted. The pH was >8.2, the test kit upper measurement limit; the pool flow meter was not operating correctly^§§§^; pool water from a large leak in the equipment room was being pumped into the pool surge pit; and no daily operational records were maintained. The splash pad and pool were closed after the investigation.

### Environmental Sampling

Environmental swab samples (from the splash pad [drains, laminar nozzles, and biofilm in the tank] and pool [gutter drains and biofilm near the pool leak]) and bulk water samples (from the splash pad tank, pool, pool leak area, and source water from a deck hose) were collected and sent to CDC’s Environmental Microbiology and Engineering Laboratory for testing using temperature-selected culture followed by molecular confirmation (*2*). On September 13, viable *N. fowleri* were detected in the swab collected from the splash pad tank. On September 14, ADH issued a press release notifying the public of a fatal case of *N. fowleri* infection and included information about the public health investigation, *N. fowleri*, and symptoms of infection.^¶¶¶^ On September 21, viable thermophilic amebas were detected in the bulk water collected from the splash pad tank and pool; *Naegleria* spp. were not detected in these bulk water samples.

### Additional Laboratory Investigation and Actions Taken

At CDC’s FLIA Laboratory, a genomic typing method using Sanger sequencing of the ITS1 loci region was conducted to compare the clinical and environmental amebas’ genetic relatedness (*3*). On September 28, the *N. fowleri* cultured from the patient’s CSF and the splash pad tank were confirmed to both be genotype III. Together, the epidemiologic, laboratory, and environmental data suggest the splash pad was the most likely site of the patient’s exposure to *N. fowleri.*

The splash pad was disabled and is no longer in use. Corrective actions**** were taken to address the code violations identified during the pool site investigation. A follow-up inspection of the pool was completed on June 3, 2024, and a new operating permit was issued.

## Discussion

*Naegleria fowleri* infections are rare and often fatal; among 164 persons known to have been infected in the United States during 1962–2023, only four (2.4%) have survived (*1*). Infection can occur when water containing the *N. fowleri* ameba enters the body through the nose (*1*). Symptoms typically begin 1–12 days after exposure; PAM progresses rapidly and can lead to brain tissue destruction, brain swelling, and death 1–18 days after symptom onset (median = 5 days) (*1*). Most *N. fowleri* infections have been associated with recreational exposure to fresh water (e.g., swimming or diving in a lake) during summer months (*1*). During 2020–2021, two children in Texas died from PAM after playing at separate “splash pads” with inadequately disinfected water: one was a single-pass, decorative fountain used as a splash pad by the public but not designed, constructed, operated, or managed as a splash pad, and the other was a splash pad that used recirculated water and was not adequately monitored (*4*,*5*). The splash pad–associated PAM case described here represents the third such case in 4 years, indicating that splash pads with inadequately disinfected water are an emerging exposure of concern for transmission of *N. fowleri*.

Clinical diagnosis of PAM is difficult because early signs and symptoms can be nonspecific, and manifestations, including CSF findings, might mimic bacterial meningitis (*6*). PAM should be considered in patients evaluated for acute meningoencephalitis who have a history of recent possible exposure to fresh water, including treated recreational water (e.g., in splash pads or pools), via nasal passages. Prompt diagnosis and treatment are critical to improving chances of survival. *N. fowleri* can be identified in CSF smears or cultures via direct visualization under a microscope using hematoxylin and eosin, periodic acid-Schiff, trichrome, Giemsa, or Wright-Giemsa stains; Gram stain is not sufficient for diagnosis, as *N. fowleri* can be destroyed during heat fixation (*7*). Confirmatory testing (PCR, immunohistochemistry, or indirect immunofluorescent staining) is available at selected U.S. laboratories, including CDC’s FLIA Laboratory^††††^ (*7*). The recommended treatment of *N. fowleri* infections is based on medications used in PAM survivors and those medications with demonstrated antiamebic activity against *N. fowleri* in the laboratory.^§§§§^

Proper design, construction, operation, and management of splash pads can help prevent transmission of pathogens, including *N. fowleri* (*8*). In this case, because no operational records were maintained and the chlorinator was reported as being nonfunctional for approximately 1 month, it is possible that inadequate disinfection of the splash pad was a chronic problem. Inadequate disinfection, operation, and management over time could lead to growth of biofilm, which can supply nutrients for free-living ameba as well as protection from disinfectants (*9*). CDC’s Model Aquatic Health Code provides evidence-based recommendations that jurisdictions and the aquatics sector can collaborate on voluntarily adopting to promote healthy swimming at splash pads, pools, and other treated recreational water venues open to the public (Box) (*10*). Healthy and safe swimming recommendations and resources for the public are also available (*4*).

BOX Selected recommendations from CDC’s Model Aquatic Health Code* to help prevent pathogen transmission, including *Naegleria fowleri*, in splash pad^†^ water^§^

- Maintain adequate disinfectant level in the water:
  - Minimum free available chlorine of 1.0 ppm (mg/L), if not using cyanuric acid (such as stand-alone cyanuric acid or stabilized chlorine, commonly known as “dichlor” or “trichlor”) (5.7.3.1.1.2.1).
  - Minimum free available chlorine of 2.0 ppm, if using cyanuric acid (5.7.3.1.1.2.2).
  - Minimum total bromine of 3.0 ppm (5.7.3.1.2.2).
- Maintain pH = 7.0–7.8 (5.7.3.4.1).
- Conduct daily inspection before opening to the public, including (6.1.2.1.5.4):
  - Ensure disinfection, secondary disinfection (e.g., ultraviolet and ozone) to inactivate pathogens further and recirculation systems and filters are operating as required.
  - Inspect for and, as needed, remove biofilm from accessible splash pad surfaces (such as the tank, spray nozzles, and drains).
- Test free available chlorine or total bromine concentration and pH before opening to the public each day and maintain adequate disinfectant concentration (5.7.5.1):
  - Test free available chlorine or total bromine and pH every 2–4 hours while open to the public (5.7.5.2– 5.7.5.3).
- Maintain water turnover times at 30 minutes or less (4.7.1.10).
- Ensure that drains prevent standing water from collecting in the water play area (4.8.1.3.1.3).
- Inspect tank regularly and, as needed, clean tank (4.12.8.6).
- Document operation and management activities, such as water testing results, response to testing results, and equipment maintenance (e.g., tank cleaning) (6.1.2.1.4.5, 6.1.2.1.5.4, and 6.4.1.2).

* https://www.cdc.gov/model-aquatic-health-code/php/our-work/index.html

^†^ A splash pad is an interactive water play venue that sprays or jets water on users.

^§^ Model Aquatic Health Code elements highlighted in this box are followed by their corresponding section numbers to facilitate referencing.

Although PAM is not a nationally notifiable disease, it is reportable in some jurisdictions, and CDC tracks *N. fowleri* infections with the voluntary assistance of health departments to monitor disease trends and guide public health recommendations.^¶¶¶¶^ Ongoing collaboration and communication among clinicians, hospitals, laboratories, CDC, health departments, the aquatics sector, and the public can support efforts to identify, treat, prevent, and control *N. fowleri* infections.

## Footnotes

* These authors contributed equally to this report.

† These senior authors contributed equally to this report.

§ CSF findings: elevated total nucleated cells (390/*μ*L [reference range = 0–5/*μ*L]) with neutrophil predominance of 71%, elevated red blood cell count (5,150/*μ*L [reference range = 0–0/*μ*L]), elevated protein (610 mg/dL [reference range = 20–70 mg/dL]), and low glucose (<4 mg/dL [reference range = 60% of a simultaneously collected blood glucose value]).

¶ 45 C.F.R. part 46, 21 C.F.R. part 56; 42 U.S.C. Sect. 241(d); 5 U.S.C. Sect. 552a; 44 U.S.C. Sect. 3501 et seq.

** Muriatic acid was reported to be used occasionally when sodium bisulfate tablets were not available on site.

†† pH was tested using a colorimetric *N,N*-diethyl-p-phenylenediamine (DPD) test kit. A maximum pH of 7.8 is permitted by Arkansas’s Rules and Regulations Pertaining to Swimming Pools and Other Related Facilities. http://170.94.37.152/REGS/007.04.12-001F-13049.pdf

§§ The residual chlorine concentration, also known as free available chlorine, was tested using a colorimetric DPD test kit. The maximum residual chlorine concentration permitted by Arkansas’s Rules and Regulations Pertaining to Swimming Pools and Other Related Facilities is 5.0 ppm. A pH >7.8 decreases how effectively residual chlorine inactivates pathogens.

¶¶ Hand-feeding chlorine can lead to inconsistencies in water disinfection. A combined high residual chlorine concentration and pH can occur after a substantial amount of chlorine is added to water, because the addition of certain chlorine products can increase pH.

*** Daily operational records would include documentation of important details, including water testing results (e.g., residual chlorine concentration and pH) and response to water testing results (e.g., what was added, how much, and when).

††† A surge tank receives gravity flow water displaced from the pool through a perimeter gutter system.

§§§ A pool flow meter, also known as a rate-of-flow indicator, is a device installed on the pool recirculation piping to indicate recirculation flow of the pool. The flow meter allows for monitoring of the turnover rate, the time it takes to run the volume of water equal to a pool’s capacity through the recirculation system, within which the water is filtered and disinfected.

¶¶¶
https://healthy.arkansas.gov/article/case-of-rare-infection-identified-in-arkansas/

**** Corrective actions included identification and repair of the large pool leak, replacement of pool flow meter, development of a new standard operating procedure (including monitoring and maintaining residual chlorine concentration and pH), and management of daily operational records. Supplementary corrective actions included installation of secondary ultraviolet radiation disinfection system and cleaning of deck drains. Live plants from planters in the pool, as well as nearby landscaping on the pool deck, were removed to help reduce loose soil and plant material contamination in the pool.

†††† Assistance with PAM diagnosis and treatment recommendations is available 24 hours a day, 7 days a week by contacting the CDC Emergency Operations Center at 770-488-7100.

§§§§
https://www.cdc.gov/naegleria/hcp/clinical-care/index.html

¶¶¶¶
https://www.cdc.gov/naegleria/programs/index.html
